# Independent influences of maternal obesity and fetal sex on maternal cardiovascular adaptation to pregnancy: a prospective cohort study

**DOI:** 10.1038/s41366-020-0627-2

**Published:** 2020-06-15

**Authors:** Noor E. W. D. Teulings, Angela M. Wood, Ulla Sovio, Susan E. Ozanne, Gordon C. S. Smith, Catherine E. Aiken

**Affiliations:** 1 Department of Public Health and Primary Care, University of Cambridge, 2 Worts’ Causeway, Cambridge, CB1 8RN Cambridge, UK; 2 Department of Obstetrics and Gynaecology, University of Cambridge, Box 223, The Rosie Hospital and NIHR Cambridge Biomedical Research Centre, Cambridge, UK; 3 University of Cambridge Metabolic Research Laboratories and MRC Metabolic Diseases Unit, Wellcome Trust-MRC Institute of Metabolic Science, Addenbrooke’s Hospital, Cambridge, CB2 0QQ UK

**Keywords:** Epidemiology, Cardiovascular diseases, Risk factors

## Abstract

**Background/Objectives:**

Successful pregnancy requires the de novo creation of low-resistance utero-placental and feto-placental circulations and incomplete remodeling of this vasculature can lead to maternal or fetal compromise. Maternal BMI and fetal sex are known to influence vascular compliance and placental development, but it is unknown if these are independent or synergistic effects. Here we aim to investigate the impact of maternal obesity, fetal sex, and any interaction thereof on maternal cardiovascular adaptation to pregnancy, by assessing the physiological drop of uterine artery doppler pulsatility (UtA-PI) and umbilical artery doppler pulsatility index (UA-PI) over gestation.

**Subjects/Methods:**

Nulliparous women with a singleton pregnancy participating in a prospective cohort study (*n* = 4212) underwent serial UtA-PI and UA-PI measurements at 20-, 28- and 36-weeks gestation. Linear mixed regression models were employed to investigate the influence of maternal BMI, fetal sex and interactions thereof on the magnitude of change in UtA-PI and UA-PI.

**Results:**

Throughout gestation, UtA-PI was higher for male fetuses and UA-PI was higher for female fetuses. The physiological drop of UtA-PI was significantly smaller in overweight (change −24.3% [95%CI −22.3, −26.2]) and obese women (change −21.3% [−18.3, −24.3]), compared to normal-weight women (change −25.7% [−24.3, −27.0]) but did not differ by fetal sex. The physiological drop in UA-PI was greater for female than male fetuses (–32.5% [−31.5, −33.5] vs. −30.7% [−29.8, −31.7]) but did not differ by maternal BMI. No interactions between maternal BMI and fetal sex were found.

**Conclusions:**

Maternal cardiovascular adaptation to pregnancy is independently associated with maternal BMI and fetal sex. Our results imply sexual dimorphism in both maternal cardiovascular adaptation and feto-placental resistance.

## Introduction

Successful pregnancy requires the de novo creation of low-resistance utero-placental and feto-placental circulations. Incomplete remodeling of the maternal spiral arteries or failure to form a sufficiently low-resistance placental circulation results in fetal and maternal compromise, and subsequent adverse outcomes, including pre-eclampsia [1–3] and fetal growth restriction [3–5]. Doppler ultrasonography can be used in pregnancy to assess the utero-placental and feto-placental circulation, with the uterine artery doppler pulsatility index (PI) reflecting vascular resistance on the maternal side of the placental circulation and the umbilical artery PI reflecting the vascular resistance on the fetal side of the placenta [6, 7].

In the non-pregnant state, obesity impairs vascular compliance and is associated with increased arterial stiffness [8, 9]. In pregnancy, high maternal BMI is associated with increased systolic blood pressure, increased left ventricular mass, and higher stroke volume [10–12], even in pregnant obese women without perinatal complications [13, 14]. Women with a higher BMI have a ‘dose-dependent’ increased risk of incomplete spiral artery conversion during pregnancy, which is likely to impair the formation of an appropriately low-resistance utero-placental circulation [15].

Fetal sex is also increasingly recognized as a key modulator of both placental development and maternal adaptation to pregnancy [16–18]. Recent evidence suggests that fetal sex differences influence the production of maternal angiogenic and fibrinolytic factors, which in turn can influence placental angiogenesis and spiral artery remodeling [19]. Broere-Brown and colleagues observed sex differences in ultrasonographic measurements of maternal vascular resistance; women pregnant with a male fetus had higher uterine artery pulsatility index (UtA-PI) in the second and third trimester compared to women carrying a female fetus [20]. Further evidence suggests that the umbilical artery pulsatility index (UA-PI) is higher in pregnancies where the fetus is female compared to male [21].

The aim of the present study was to define the longitudinal impact of maternal BMI and fetal sex on resistance in the utero-placental and feto-placental circulation, and in particular whether these are synergistic or independent factors. Crucially, both of these factors can vary between different pregnancies in the same woman. BMI changes between subsequent pregnancies are relatively common and alter the risk of an adverse pregnancy outcome [22]. Fetal sex is determined as-if-at-random for each pregnancy and is also an important influence on pregnancy success [23, 24]. Our findings may therefore help to explain variability in pregnancy complications experienced by women in successive pregnancies.

## Subjects and methods

### Study design

The Pregnancy Outcome Prediction (POP) study was a prospective cohort study of nulliparous primiparous women attending the Rosie Hospital, Cambridge (UK) for their dating scan (11–14 weeks) between January 14, 2008 and July 31, 2012. Women with a viable singleton pregnancy were eligible for inclusion. The study protocol has previously been described in detail [25]. Ethical approval for the study was obtained from the Cambridgeshire 2 Research Ethics Committee (reference number 07/H0308/163). Written informed consent was given by all participants. Women participating in this study underwent serial research ultrasound scans at 20, 28, and 36 weeks of gestation, with both the woman and the clinician blinded to the result of the scans.

### Doppler measurements

The uterine artery pulsatility index (UtA-PI) was assumed to reflect the resistance in the utero-placental circulation and thus the efficacy of spiral artery remodeling [1], with a higher UtA-PI indicating narrow and stiff spiral arteries [26]. The umbilical artery pulsatility index (UA-PI) was assumed to primarily reflect resistance in the feto-placental circulation (although it will also depend on fetal cardiac function [27, 28]).

### Definitions

Body mass index (BMI) was calculated from maternal weight and height measured on the day of the booking scan and was used as proxy for pre-pregnancy BMI. Maternal BMI categories were based on the WHO BMI categories, with BMI < 18.5 classified as underweight, BMI 18.5–24.99 as normal weight, BMI 25–29.99 as overweight and BMI ≥ 30 as obese. Research ultrasound scans were conducted at 20, 28 and 36 weeks of gestation. The management and analysis of the ultrasound data have been described in detail previously [29]. Maternal age was defined as age at recruitment. Ethnicity was self-reported via a questionnaire at the 20-week scan. Gestational age was based on the estimation at time of the first scan. The cut off values for clinically relevant reference ranges of the PI indices used were (i) uterine artery PI > 95th centile at 20 wkGA (ii) umbilical artery PI > 95th centile at 28 wkGA and (iii) umbilical artery PI > 95th centile at 36 wkGA.

### Data analysis

Linear mixed regression analyses were used to model the repeat absolute log-transformed UtA-PI or UA-PI measurements to assess the changes in UtA-PI or UA-PI levels over gestation. The linear mixed models included a random intercept per woman, fixed effects for BMI category and/or fetal sex (and interactions thereof) at the estimated gestational age at each research scanning time (i.e., 20, 28 or 36 weeks). Further adjustment was made for maternal systolic blood pressure measured at 12 weeks, maternal ethnicity, maternal age, marital status, maternal smoking status and deprivation index. Covariates were selected based on clinical relevance. The model used to estimate the unadjusted log-transformed levels of UtA-PI over gestation by BMI categories can be represented as:

$$\log \left( {UtA - PI_{ij}} \right) = NORMAL\,WEIGHT_i \times \mathop {\sum}\limits_{j = 1}^3 {\beta _{0j}d_{ij} + OVERWEIGHT_i} \times \mathop {\sum}\limits_{j = 1}^3 {\beta _{1j}d_{ij} + OBESE_i} \times \mathop {\sum}\limits_{j = 1}^3 {\beta _{2j}d_{ij}}$$

where *d*_*i1*_ equals the estimated gestational age at the 20-week scan minus 20, *d*_*i2*_ represents the estimated gestational age at the 28-week minus 28 and *d*_*i3*_ represents the estimated gestational age at the 36-week minus 36; *u*_*i*_ represents the random intercept and *e*_*ij*_ represents residual error for individual *i* and scanning time *j* for *j* = 1,…,3. This parameterization allows easy interpretation, for example, *β*_11_, *β*_12_ and *β*_13_ represent the log-transformed values in UtA-PI at exactly 20, 28 or 36 weeks, respectively for normal-weight women.

As a sensitivity analysis, we repeated the analyses to (i) include glucose measurement at 28 weeks, (ii) add gestational weight gain as covariates and (iii) exclude women who experienced any perinatal complication during pregnancy (gestational diabetes, preeclampsia, gestational hypertension or preterm birth).

Statistical analyses were performed using R [30] with the lme4 package [31] for performing mixed linear models. Figures were produced using the ggplot2 package [32].

## Results

**Fig. 1:**
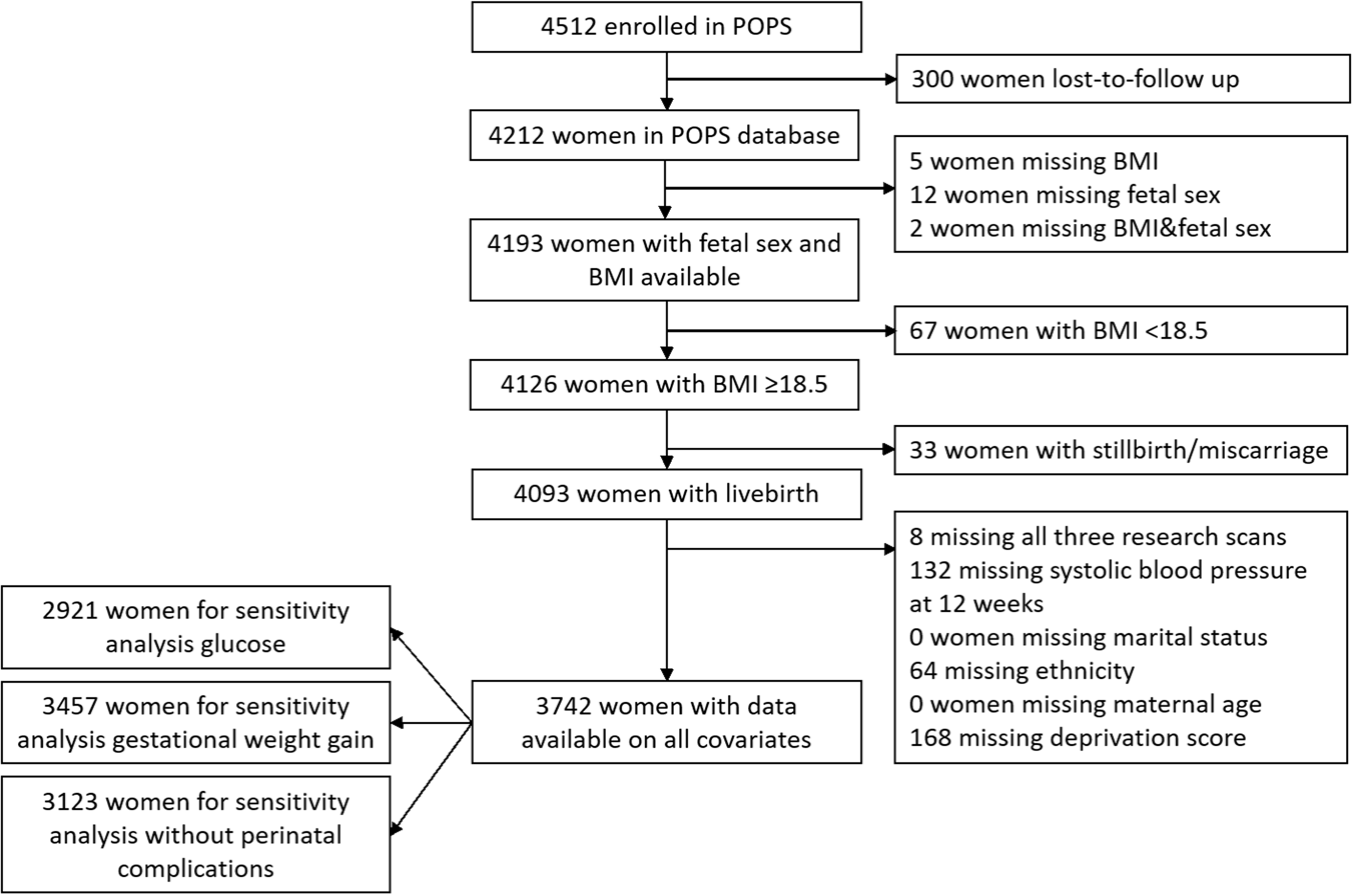
Flow diagram showing in- and exclusion criteria for study into the influence of maternal obesity and fetal sex on cardiovascular adaptation to pregnancy in the POPS cohort. The final population available for analysis was 3742 women. POPS; Pregnancy Outcome Prediction Study, BMI Body Mass Index.

A total of 4512 women participated in the POP study, with 300 women lost to follow up. For this analysis, we excluded women with missing information on maternal BMI and/or fetal sex (*n* = 19), with a stillbirth or miscarriage (*n* = 33), with missing covariates (*n* = 351) and who were underweight (*n* = 67), since the underweight group was underpowered for inference (Fig. 1). A total of 3742 women were included in the analyses, of whom 57.8% had a normal weight (BMI 18.5–24.9 kg/m^2^), 28.3% were overweight (BMI 25.0–29.9 kg/m^2^), and 13.9% were classified as obese (BMI ≥ 30.0 kg/m^2^) (Table 1). Women with higher maternal BMI were more likely to be smokers, and have pre-existing and gestational hypertension, as well as preeclampsia and gestational diabetes. Neonates born to overweight and obese women were more likely to have higher birthweight and placental weight compared to babies born to normal-weight women (Table 1). There were no significant differences in maternal baseline characteristics or perinatal complications between fetal sexes. Male neonates had on average about 125 g higher birthweight compared to female neonates (Supplementary Table 1).

**Table 1:** Baseline and birth characteristics stratified by maternal BMI category.

	Normal weight	Overweight	Obese	Total
	*n* = 2164	*n* = 1059	*n* = 519	*n* = 3742
Maternal age (y)	30.0 (4.8)	30.4 (5.3)	29.3 (5.7)	30.0 (5.1)
Gestational age at first scan	12.6 (0.8)	12.6 (0.8)	12.6 (0.9)	12.6 (0.9)
Gestational weight gain (kg)^a^				
Total weight gain	12.4 (3.7)	12.8 (4.5)	10.8 (5.4)	12.3 (4.2)
From 12 to 20 weeks	3.4 (2.1)	3.2 (2.2)	2.6 (2.4)	3.2 (2.2)
From 20 to 28 week	4.7 (2.2)	4.9 (2.3)	4.0 (2.3)	4.6 (2.3)
From 28 to 36 weeks	4.3 (2.3)	4.6 (2.4)	4.2 (2.7)	4.4 (2.4)
Smoking status				
Non-smoker	1381 (63.8%)	592 (55.9%)	266 (51.3%)	2239 (59.8%)
Quit pre-pregnancy	536 (24.8%)	330 (31.2%)	161 (31.0%)	1027 (27.4%)
Quit during pregnancy	143 (6.6%)	93 (8.8%)	58 (11.2%)	294 (7.9%)
Current smokers	104 (4.8%)	44 (4.2%)	34 (6.6%)	182 (4.9%)
Systolic BP (12 wkGA (mmHg))	106.5 (11)	109.4 (11)	114.5 (11)	108.4 (11)
Fetal sex				
Male	1090 (50.4%)	531 (50.1%)	264 (50.9%)	1885 (50.4%)
Female	1074 (49.6%)	528 (49.9%)	255 (49.1%)	1857 (49.6%)
Ethnicity				
White	2027 (93.7%)	1005 (94.9%)	498 (96.0%)	3530 (94.3%)
Other	137 (6.3%)	54 (5.1%)	21 (4.0%)	212 (5.7%)
Marital status				
Married	1535 (70.9%)	718 (67.8%)	316 (60.9%)	2569 (68.7%)
Not married	629 (29.1%)	341 (32.2%)	203 (39.1%)	1173 (31.3%)
Deprivation score				
1 (lowest)	555 (25.6%)	282 (26.6%)	112 (21.6%)	949 (25.4%)
2	518 (23.9%)	276 (26.1%)	128 (24.7%)	922 (24.6%)
3	541 (25.0%)	271 (25.6%)	132 (25.4%)	944 (25.2%)
4 (highest)	550 (25.4%)	230 (21.7%)	147 (28.3%0	927 (24.8%)
Pre-existing diabetes				
Yes	4 (0.2%)	10 (0.9%)	2 (0.4%)	16 (0.4%)
No	2160 (99.8%)	1049 (99.1%)	517 (99.6%)	3726 (99.6%)
Pre-existing hypertension				
Yes	64 (3.0%)	68 (6.4%)	67 (12.9%)	199 (5.3%)
No	2100 (97.0%)	991 (93.6%)	452 (87.1%)	3543 (94.7%)
Gestational hypertension				
Yes	24 (1.1%)	23 (2.2%)	20 (3.9%)	67 (1.8%)
No	2138 (98.8%)	1035 (97.7%)	499 (96.1%)	3672 (98.1%)
Unknown	2 (0.1%)	1 (0.1%)	0 (0.0%)	3 (0.1%)
Preeclampsia				
Yes	95 (4.4%)	76 (7.2%)	84 (16.2%)	255 (6.8%)
No	2067 (95.5%)	982 (92.7%)	435 (83.8%)	3484 (93.1%)
Unknown	2 (0.1%)	1 (0.1%)	0 (0.0%)	3 (0.1%)
Gestational diabetes				
Yes	58 (2.7%)	62 (5.9%)	57 (11.0%)	177 (4.7%)
No	2105 (97.3%)	993 (93.8%)	462 (89.0%)	3560 (95.1%)
Unknown	1 (0.0%)	4 (0.4%)	0 (0.0%)	5 (0.1%)
Birthweight (g)	3380 (498)	3444 (519)	3498 (569)	3414 (516)
Placental weight (g)	451 (93)	473 (102)	490 (489)	463 (99)

Data are represented as mean (SD) or as number (%). Differences in baseline characteristics were tested using chi-square tests and Kruskal–Wallis tests.

*BP* blood pressure.

^a^n-number for gestational weight gain at (i) 12–20wk; normal weight 2139, overweight 1048 and obese 515, (ii) 20–28wk; normal weight 2085, overweight 1024 and obese 505, (iii) 28–36 wk; normal weight 1991, overweight 955 and obese 465, (iv) 12–36wk; normal weight 2021, overweight 965 and obese 471.

**Fig. 2:**
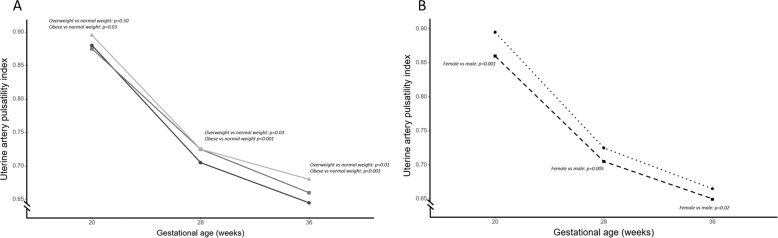
Physiological drop in uterine artery pulsatility index over the course of gestation. **a** Development of uterine artery pulsatility index (UtA-PI) over gestation stratified by maternal BMI category. Absolute values at scanning timepoints [median (IQR)]; normal weight-women; 20 wks [0.88 (0.72–1.09)], 28 wks [0.71 (0.61–0.84)], 36 wks [0.65 (0.56–0.77). Overweight women; 20 wks [0.88 (0.73–1.08)], 28 wks [0.73 (0.62–0.85)], 36 wks [0.66 (0.56–0.78)]. Obese women; 20wks [0.90 (0.74–1.09)], 28 wks [0.73 (0.63–0.88)], 36 wks [0.68 (0.59–0.82)]. Black; women with normal weight, dark gray; overweight women, light gray; obese women. **b** Development of UtA-PI over gestation stratified by fetal sex. Absolute values at scanning timepoints [median (IQR)]; male fetuses; 20 wks [0.90 (0.74–1.10)], 28 wks [0.73 (0.62–0.86)], 36 wks [0.67 (0.58–0.78)]. Female fetuses; 20 wks [0.86 (0.71–1.07)], 28wks [0.71 (0.60–0.84)], 36 wks [0.65 (0.55–0.77)]. Dashed line; women carrying female fetus, dotted line; women carrying male fetus. Estimates are simple observed medians at each scanning time, statistics shown are from mixed linear model corrected for gestational age. All *p* values are compared to normal-weight women at same time period or women carrying a male fetus at the same time period.

The absolute values of UtA-PI were similar between normal, overweight and obese women at the 20-week scan (Fig. 2a). The physiological drop in UtA-PI between 20 and 36 weeks was lower in obese women compared to women of normal weight (mean drop −21.3% [95%CI −18.3, −24.2] vs −25.7% [−24.3, −27.0], respectively, *p* < 0.001) (Table 2), which remained after correction for maternal variables including maternal BMI, systolic blood pressure at 12 weeks gestation, marital status, maternal age, maternal ethnicity and deprivation index. This overall decrease in physiological drop is due to a diminished fall in resistance in the early phase (20–28 weeks), rather than the later phase (28–36 weeks).

**Table 2:** Percentage change in uterine artery pulsatility index over the course of gestation by maternal BMI category, expressed as percentage drop of Doppler PI between scanning timepoints.

	Drop between 20- and 36-week scan				Drop between 20- and 28-week scan				Drop between 28- and 36-week scan			
	Model 1^a^		Model 2^b^		Model 1^a^		Model 2^b^		Model 1^a^		Model 2^b^	
	Percentage decrease [95 % CI]	*p* value^c^	Percentage decrease [95 % CI]	*p* value^c^	Percentage decrease [95 % CI]	*p* value^c^	Percentage decrease [95 % CI]	*p* value^c^	Percentage decrease [95 % CI]	*p* value^c^	Percentage decrease [95 % CI]	*p* value^c^
Normal weight (*n* = 2164)	−25.7% [−24.3, −27.0]	ref	−25.7% [−24.3, −27.0]	ref	−19.7% [−18.3, −21.0]	ref	−19.7% [−18.3, −21.0]	ref	−7.4% [−6.1, −8.8]	ref	−7.4% [−6.1, −8.8]	ref
Overweight (*n* = 1059)	−24.3% [−22.3, −26.2]	0.13	−24.3% [−22.3, −26.3]	0.13	−17.9% [−16.0, −19.8]	0.07	−17.9% [−16.0, −19.8]	0.07	−7.7% [−5.8, −9.7]	0.79	−7.7% [−5.8, −9.7]	0.79
Obese (*n* = 519)	−21.3% [−18.3, −24.3]	<0.001	−21.3% [−18.3, −24.2]	<0.001	−16.4% [−13.5, −19.3]	0.01	−16.4% [−13.5, −19.3]	0.01	−5.8% [−2.8, −8.8]	0.30	−5.8% [−2.8, −8.8]	0.30

*CI* Confidence Interval.

^a^Model adjusted for gestational age at all scanning timepoints.

^b^Model adjusted for gestational age at all scanning timepoints, maternal BMI, systolic blood pressure at 12 weeks gestation, marital status, maternal age, maternal ethnicity, maternal smoking status and deprivation index.

^c^*p* value relative to mean uterine artery pulsatility index drop in normal-weight women at same scanning timepoint.

UtA-PI was higher throughout gestation for women carrying a male versus female fetus (Fig. 2b). Fetal sex did not significantly influence the magnitude of the drop in uterine artery PI over gestation (Supplementary Table 2). There was no evidence of an interaction between fetal sex and maternal BMI on UtA-PI over gestation (Supplementary Table 3).

**Fig. 3:**
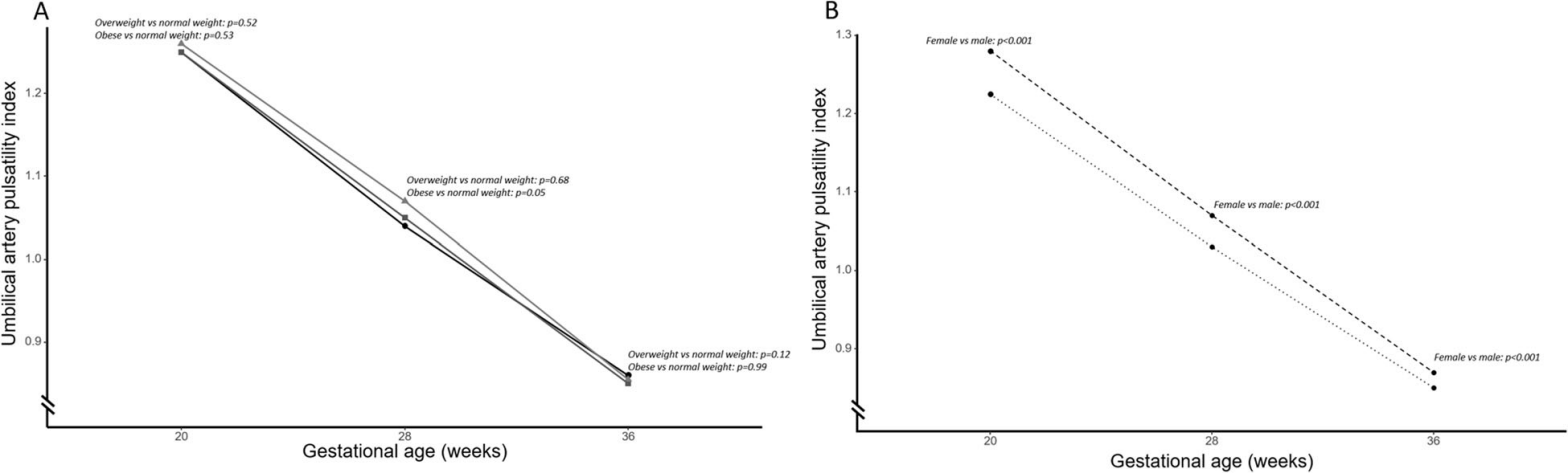
Physiological drop in the umbilical artery pulsatility index over the course of gestation. **a** Development of umbilical artery pulsatility index (UA-PI) over gestation stratified by maternal BMI category. Absolute values at scanning timepoints [median (IQR)]; normal-weight women; 20 wks [1.25 (1.13–1.38)], 28 wks [1.04 (0.93–1.16)], 36 wks [0.86 (0.76–0.97)]. Overweight women; 20 wks [1.25 (1.14–1.37)], 28 wks [1.05 (0.94–1.16)], 36 wks [0.85 (0.74–0.97)]. Obese women; 20wks [1.26 (1.13–1.37)], 28 wks [1.07 (0.95–1.18)], 36 wks [0.85 (0.75–0.96)]. Black; women with normal weight, dark gray; overweight women, light gray; obese women. **b** Development of UA-PI over gestation stratified by fetal sex. Absolute values at scanning timepoints [median (IQR)]; male fetuses; 20wks [1.22 (1.11–1.34)], 28wks [1.03 (0.92–1.14)], 36 wks [0.85 (0.75–0.96)]. Female fetuses; 20 wks [1.28 (1.16–1.40)], 28 wks [1.07 (0.96–1.20)], 36 wks [0.87 (0.76–0.98)]. Dashed line; women carrying female fetus, dotted line; women carrying male fetus. Estimates are simple observed medians at each scanning time, statistics shown are from mixed linear model corrected for gestational age. All *p* values are compared to normal-weight women at same time period or women carrying a male fetus at the same time period.

Women carrying a female fetus had a higher UA-PI throughout all of gestation compared to women carrying a male fetus (Fig. 3b). The overall drop in UA-PI between 20 and 36 weeks was greater in women carrying a female fetus compared to a male fetus (−32.5% [−31.5, −33.5] vs −30.7% [−29.8, −31.7], respectively, *p* < 0.001) (Table 3). We did not observe any differences in umbilical artery pulsatility indices or the drop in UA-PI between maternal BMI categories (Fig. 3a and Supplementary Table 4). We did not find any sexual dimorphism in the relationship between UA-PI and maternal pre-pregnancy BMI (Supplementary Table 5).

**Table 3:** Percentage change in umbilical artery pulsatility index over the course of gestation by fetal sex, expressed as percentage drop of Doppler PI between scanning timepoints.

	Drop between 20- and 36-week scan				Drop between 20- and 28-week scan				Drop between 28- and 36-week scan			
	Model 1^a^		Model 2^b^		Model 1^a^		Model 2^b^		Model 1^a^		Model 2^b^	
	Percentage decrease [95 % CI]	*p* value^c^	Percentage decrease [95 % CI]	*p* value^c^	Percentage decrease [95 % CI]	*p* value^c^	Percentage decrease [95 % CI]	*p* value^c^	Percentage decrease [95 % CI]	*p* value^c^	Percentage decrease [95 % CI]	*p* value^c^
Male fetus (*n* = 1885)	−30.7% [−29.8, −31.7]	ref	−30.7% [−29.8, −31.7]	ref	−16.4% [−15.4, −17.3]	ref	−16.4% [−15.4, −17.3]	ref	−17.2% [−16.2, −18.2]	ref	−17.2% [−16.2, −18.2]	ref
Female fetus (*n* = 1857)	−32.5% [−31.5, −33.5]	<0.001	−32.5% [−31.5, −33.5]	<0.001	−16.8% [−15.8, −17.7]	0.46	−16.8% [−15.8, −17.7]	0.46	−18.9% [−17.9, −19.8]	0.004	−18.9% [−17.9, −19.8]	0.004

*CI* Confidence Interval.

^a^Model adjusted for gestational age at all scanning timepoints.

^b^Model adjusted for gestational age at all scanning timepoints, maternal BMI, systolic blood pressure at 12 weeks gestation, marital status, maternal age, maternal ethnicity, maternal smoking status and deprivation index.

^c^*p* value relative to mean umbilical artery pulsatility index drop in women carrying a male fetus at same scanning timepoint.

A total of 5% of women had a UtA-PI value >95th centile at 20 wkGA; 5.3% of normal-weight women were above the reference range, while 4.9% of overweight and 4.0% of obese women had UtA-PI values above the 95th centile at 20 wkGA (chi-square test *p* value = 0.50). At 28 wkGA, 5.0% of women had a UA-PI value about the 95th centile; 4.4% of the women carrying a male fetus and 5.6% of the women carrying a female fetus were above the reference range (chi-square test *p* value = 0.28). The same pattern was seen at 36 wkGA for a UA-PI value >95th centile; 4.7% of all women, 4.3% of women carrying a male fetus and 5.1% of women carrying a female fetus had a UA-PI > 95th centile at 36 wkGA (chi-square test *p* value = 0.78).

Results for the sensitivity analysis investigating the influence of glucose levels at 28 weeks gestation or gestational weight gain, as well as excluding women with gestational diabetes, preeclampsia, gestational hypertension and preterm birth were unchanged from the main analysis (Supplementary Tables 6–9).

## Discussion

### Main findings

We assessed the impact of maternal obesity and fetal sex on both utero-placental and feto-placental resistance over the course pregnancy, with the aim of improving understanding of the variability in risk of complications in successive pregnancies. We show that resistance in the utero-placental circulation is independently influenced by both maternal BMI and fetal sex. The physiological drop in uterine artery PI over the course of gestation was attenuated in women who were overweight or obese compared to women whose BMI was in the normal range. The impact of maternal BMI on utero-placental resistance became greater as the pregnancy progressed. By contrast, the impact of fetal sex on utero-placental resistance was consistent throughout gestation. Women carrying a male fetus had consistently higher uterine artery doppler PI compared to women carrying female fetuses at every measured time-point.

Resistance in the feto-placental circulation was independent of maternal BMI but influenced by fetal sex. Pulsatility index in the umbilical artery was higher in women carrying a female fetus compared to women carrying a male fetus at all time-points, but the magnitude of difference between sexes reduced with increasing gestation.

Previous studies have shown similar patterns when investigating fetal sex differences in the absolute values of UtA-PI in the second and third trimester [33], and between maternal pre-pregnancy BMI and higher UtA-PI in the third trimester [34]. However, in contrast to our study, prior research did not assess the physiological change in vascular resistance over the course of gestation or a possible interaction between maternal BMI and fetal sex.

### Strengths and limitations

A major strength of the current work is the detailed phenotyping and completeness of the data available regarding pregnancies in the POPS cohort [35]. In particular, longitudinal ultrasonographic measurements of both the uterine and umbilical artery pulsatility indices from 20 weeks of pregnancy through to 36 weeks are available on a large cohort of nulliparous women. Moreover, the detailed set of covariates in the POPS dataset allowed adjustment of our models for other relevant maternal characteristics. A limitation of our study is the lack of availability of other Doppler parameters (e.g., resistance index) as well as the lack of longitudinal blood pressure data, as previous studies have shown a significant effect of maternal weight [36, 37] as well as fetal sex [20] on the systolic and diastolic blood pressure.

### Interpretation

The adverse impact of higher maternal BMI on utero-placental, but not feto-placental, vascular resistance during pregnancy may reflect reduced baseline compliance in the uterine (and other systemic) vasculature in women with high BMI [8, 38]. Remodeling of the uterine vasculature is one of the major changes required to provide adequate utero-placental perfusion to facilitate fetal growth [39]. A failure to facilitate an adequate drop in the UtA-PI is related to an increased incidence of placenta-related diseases in the third trimester [40]. There is extensive literature suggesting that obesity impairs nitric oxide (NO) availability [reviewed in [41–43]], which could reduce pregnancy-induced NO-mediated vasodilation [44, 45], thus impairing the expected physiological drop in vascular resistance during pregnancy in women with higher BMIs. A further possibility is that spiral artery conversion, necessary to enable a low-resistance vascular bed for exchange of nutrients and oxygen to the fetus, is more likely to be incomplete in women with higher BMI. Adequate spiral artery remodeling, via extravillous trophoblast invasion, is required to facilitate the normal physiological drop in utero-placental vascular resistance over the course of gestation [46]. Spiral artery remodeling is initiated by uterine natural killer cells, which exhibit functional changes in gene expression and growth factor signaling when exposed to maternal obesity [47]. Higher BMI may therefore be associated with incomplete spiral artery remodeling, and hence increased utero-placental vascular resistance throughout gestation.

Few previous studies have examined differences in utero-placental vascular resistance by fetal sex. Widnes and colleagues reported no differences in uterine artery resistance at 22–24 weeks gestation between fetal sexes [21], whereas, Broere-Brown reported a systemically higher UtA-PI in the second and third trimester in women carrying a male fetus [33], consistent with the our findings. The mechanism by which fetal sex can influence pulsatility index in the uterine artery is not known, however, placental sex has a profound effect on the placental transcriptome, largely mediated by genes which escape from X chromosome inactivation. These differences include both previously recognized and placental-specific escapees and these changes in turn alter the maternal serum metabolome [17]. Hence, it is plausible that fetal sex may alter maternal cardiovascular adaptation to pregnancy [48]. However, there are also morphological differences in male versus fetal placentas, for example placental weight, capillary density and trophoblast differentiation [49, 50], which may be reflected in a direct difference in placental vascular resistance affecting flow in both the utero-placental and feto-placental circulations.

## Conclusions

Our analysis implies that higher BMI and male fetal sex are independent risk factors for higher resistance in the uterine artery, which act through distinct pathways. Previous reports suggest that there is a higher incidence of placenta-mediated pathologies, for example pre-eclampsia, in pregnancies with male rather than female fetuses [51], which could be linked to the observed differences in utero-placental and feto-placental blood flow. Furthermore, maternal pre-pregnancy BMI is also a known risk factor for pathologies linked to impaired utero-placental blood flow, for example IUGR and pre-eclampsia [52, 53]. Our finding that higher maternal BMI is associated with attenuation of the physiological drop in pulsatility index of the uterine artery across gestation provides impetus for further work exploring interventions that can improve utero-placental blood flow in mothers with higher BMI. Pulsatility index in the uterine artery is a key predictor of adverse pregnancy outcomes, such as preeclampsia and fetal growth restriction [2, 54], hence our findings give new insight into the independent risks posed by high maternal BMI and male fetal sex.

## Supplementary information

Supplementary table 1

Supplementary table 2

Supplementary table 3

Supplementary table 4

Supplementary table 5

Supplementary table 6

Supplementary table 7

Supplementary table 8

Supplementary table 9
