# Supplementary material for: Independent influences of maternal obesity and fetal sex on maternal cardiovascular adaptation to pregnancy: a prospective cohort study
Source: Int J Obes (Lond). 2020 Jun 15;44(11):2246–55. doi: 10.1038/s41366-020-0627-2 (PMC7577853; doi:10.1038/s41366-020-0627-2)
Supplement: Supplementary file 1 — Supplementary table 1 [file 41366_2020_627_MOESM1_ESM.docx]

|  |  | Male fetus | Female fetus | Total |
| --- | --- | --- | --- | --- |
|  |  | n = 1885 | n = 1857 | n = 3742 |
| Maternal age (y) | | 30.1 (5.1) | 29.9 (5.1) | 30.0 (5.1) |
| Gestational age at first scan | | 12.7 (0.8) | 12.6 (0.9) | 12.6 (0.9) |
| Gestational weight gain (kg)* | |  |  |  |
|  | Total weight gain | 12.4 (4.3) | 12.1 (4.1) | 12.3 (4.2) |
|  | From 12 to 20 weeks | 3.3 (2.2) | 3.2 (2.1) | 3.2 (2.2) |
|  | From 20 to 28 weeks | 4.7 (2.3) | 4.6 (2.2) | 4.6 (2.3) |
|  | From 28 to 36 weeks | 4.4 (2.4) | 4.3 (2.3) | 4.4 (2.4) |
| Smoking status | |  |  |  |
|  | Non-smoker | 1151 (61.1%) | 1088 (58.6%) | 2239 (59.8%) |
|  | Quit pre-pregnancy | 486 (25.8%) | 541 (29.1%) | 1027 (27.4%) |
|  | Quit during pregnancy | 149 (7.9%) | 145 (7.8%) | 294 (7.9%) |
|  | Current smokers | 99 (5.3%) | 83 (4.5%) | 182 (9.8%) |
| Systolic BP (12 wkGA (mmHg)) | | 108.5 (12) | 108.3 (11) | 108.4 (11) |
| Maternal BMI category | |  |  |  |
|  | Normal weight (18.5-24.9kg/m^2^) | 1090 (57.8%) | 1074 (57.8%) | 2164(57.8%) |
|  | Overweight (25-29.9 kg/m^2^) | 531 (28.2%) | 528 (28.4%) | 1059 (57.0%) |
|  | Obese (>30 kg/m^2^) | 264 (14.0%) | 255 (13.7%) | 519 (27.9%) |
| Ethnicity | |  |  |  |
|  | White | 1773 (94.1%) | 1757 (94.6%) | 3530 (94.3%) |
|  | Other | 112 (5.9%) | 100 (5.4%) | 212 (5.7%) |
| Marital status | |  |  |  |
|  | Married | 1297 (68.8%) | 1272 (68.5%) | 2569 (68.7%) |
|  | Not married | 588 (31.2%) | 585 (31.5%) | 1173 (31.3%) |
| Deprivation score | |  |  |  |
|  | 1 (lowest) | 495 (26.3%) | 454 (24.4%) | 949 (25.4%) |
|  | 2 | 452 (24.0%) | 470 (25.3%) | 922 (24.6%) |
|  | 3 | 465 (24.7%) | 479 (25.8%) | 944 (25.2%) |
|  | 4 (highest) | 473 (25.1%) | 454 (24.4%) | 927 (24.8%) |
| Pre-existing diabetes | |  |  |  |
|  | Yes | 7 (0.4%) | 9 (0.5%) | 16 (0.4%) |
|  | No | 1878 (99.6%) | 1848 (99.5%) | 3726 (99.6%) |
| Pre-existing hypertension | |  |  |  |
|  | Yes | 106 (5.6%) | 93 (5.0%) | 199 (5.3%) |
|  | No | 1779 (94.4%) | 1764 (95.0%) | 3543 (94.7%) |
| Gestational hypertension | |  |  |  |
|  | Yes | 35 (1.9%) | 32 (1.7%) | 67 (1.8%) |
|  | No | 1848 (98.0%) | 1824 (98.2%) | 3672 (98.1%) |
|  | Unknown | 2 (0.1%) | 1 (0.1%) | 3 (0.1%) |
| Preeclampsia | |  |  |  |
|  | Yes | 138 (7.3%) | 117 (6.3%) | 255 (6.8%) |
|  | No | 1745 (92.6%) | 1739 (93.6%) | 3484 (93.1%) |
|  | Unknown | 2 (0.1%) | 1 (0.1%) | 3 (0.1%) |
| Gestational diabetes | |  |  |  |
|  | Yes | 96 (5.1%) | 81 (4.4%) | 177 (4.7%) |
|  | No | 1786 (94.7%) | 1774 (95.5%) | 3560 (95.1%) |
|  | Unknown | 3 (0.2%) | 2 (0.1%) | 5 (0.1%) |
| Birthweight (g) | | 3477 (529) | 3350 (494) | 3414 (516) |
| Placental weight (g) | | 465 (99) | 461 (99) | 463 (99) |

**Supplementary table 1: Baseline and birth characteristics stratified by fetal sex.** Data are represented as mean (SD) or as number (%). BP; blood pressure. Differences in baseline characteristics were tested using chi-square tests and Kruskal-Wallis tests. * n-number for gestational weight gain at (i) 12-20wk; male fetus 1866, female fetus 1836 (ii) 20-28wk; male fetus 1814, female fetus 1800, (iii) 28-36 wk; male fetus 1709, female fetus 1702, (iv) 12-36wk; male fetus 1732, female fetus 1725.
