# Supplementary material for: Independent influences of maternal obesity and fetal sex on maternal cardiovascular adaptation to pregnancy: a prospective cohort study
Source: Int J Obes (Lond). 2020 Jun 15;44(11):2246–55. doi: 10.1038/s41366-020-0627-2 (PMC7577853; doi:10.1038/s41366-020-0627-2)
Supplement: Supplementary file 2 — Supplementary table 2 [file 41366_2020_627_MOESM2_ESM.docx]

|  | Drop between 20- and 36-week scan | | | | Drop between 20- and 28-week scan | | | | Drop between 28- and 36-week scan | | | |
| --- | --- | --- | --- | --- | --- | --- | --- | --- | --- | --- | --- | --- |
|  | Model 1^a^ | | Model 2^b^ | | Model 1^a^ | | Model 2^b^ | | Model 1^a^ | | Model 2^b^ | |
|  | Percentage decrease  [95 % CI] | p value^c^ | Percentage decrease  [95 % CI] | p value^c^ | Percentage decrease  [95 % CI] | p value^c^ | Percentage decrease  [95 % CI] | p value^c^ | Percentage decrease  [95 % CI] | p value^c^ | Percentage decrease  [95 % CI] | p value^c^ |
| Male fetus  (n=1885) | -24.9%  [-23.5, -26.4] | Ref | -24.9%  [-23.5, -26.4] | ref | -19.1%  [-17.6, -20.5] | ref | -19.1%  [-17.6, -20.6] | ref | -7.2%  [-5.7, -8.7] | ref | -7.2%  [-5.7, -8.7] | ref |
| Female fetus  (n=1857) | -24.5%  [-23.0, -26.0] | 0.57 | -24.5%  [-23.0, -26.0] | 0.55 | -18.4%  [-16.9, -19.9] | 0.43 | -18.4%  [-16.9, -19.9] | 0.42 | -7.5%  [-6.0, -9.0] | 0.83 | -7.5%  [-5.9, -8.9] | 0.84 |

**Supplementary table 2: Percentage change in uterine artery pulsatility index over the course of gestation by fetal sex, expressed as percentage drop of Doppler PI between scanning timepoints.** CI; Confidence Interval. ^a^Model adjusted for gestational age at all scanning timepoints ^b^Model adjusted for gestational age at all scanning timepoints, maternal BMI, systolic blood pressure at 12 weeks gestation, marital status, maternal age, maternal ethnicity, maternal smoking status and deprivation index. ^c^p-value relative to mean uterine artery pulsatility index drop in normal weight women at same scanning timepoint.
