# Supplementary material for: Independent influences of maternal obesity and fetal sex on maternal cardiovascular adaptation to pregnancy: a prospective cohort study
Source: Int J Obes (Lond). 2020 Jun 15;44(11):2246–55. doi: 10.1038/s41366-020-0627-2 (PMC7577853; doi:10.1038/s41366-020-0627-2)
Supplement: Supplementary file 3 — Supplementary table 3 [file 41366_2020_627_MOESM3_ESM.docx]

|  |  | Drop between 20- and 36-week scan | | | | Drop between 20- and 28-week scan | | | | Drop between 28- and 36-week scan | | | |
| --- | --- | --- | --- | --- | --- | --- | --- | --- | --- | --- | --- | --- | --- |
|  |  | Model 1^a^ | | Model 2^b^ | | Model 1^a^ | | Model 2^b^ | | Model 1^a^ | | Model 2^b^ | |
|  |  | Percentage decrease  [95 % CI] | p value^c^ | Percentage decrease  [95 % CI] | p value^c^ | Percentage decrease  [95 % CI] | p value^c^ | Percentage decrease  [95 % CI] | p value^c^ | Percentage decrease  [95 % CI] | p value^c^ | Percentage decrease  [95 % CI] | p value^c^ |
| Male fetus | Normal weight  (n=1090) | -25.7%  [-23.8, -27.6] | ref | -25.7%  [-23.8, -27.6] | ref | -20.1%  [-18.2, -22.0] | ref | -20.1%  [-18.2, -22.0] | ref | -7.1%  [-5.1, -9.0] | ref | -7.1%  [-5.1, -9.0] | ref |
|  | Overweight  (n=531) | -25.4%  [-22.6, -28.2] | 0.79 | -25.4%  [-22.6, -28.2] | 0.79 | -18.2%  [-15.4, -20.9] | 0.17 | -18.2%  [-15.5, -20.9] | 0.17 | -8.8%  [-6.0, -11.6] | 0.28 | -8.8%  [-6.0, -11.6] | 0.28 |
|  | Obese  (n=264) | -20.3%  [-16.2, -24.5] | 0.002 | -20.3%  [-16.1, -24.5] | 0.002 | -16.6%  [-12.5, -20.7] | 0.06 | -16.5%  [-12.4, -20.6] | 0.06 | -4.5%  [-0.3, -8.7] | 0.24 | -4.5%  [-0.3, -8.7] | 0.24 |
| Female fetus | Normal weight  (n=1074) | -25.5%  [-23.6, -27.5] | ref | -25.6%  [-23.6, -27.5] | Ref | -19.2%  [-17.3, -21.2] | ref | -19.2%  [-17.3, -21.2] | ref | -7.8%  [-5.9, -9.8] | ref | -7.8%  [-5.9, -9.8] | ref |
|  | Overweight  (n=528) | -23.1%  [-20.3, -26.0] | 0.06 | -23.1%  [-20.3, -26.0] | 0.06 | -17.6%  [-14.9, -20.4] | 0.24 | -17.6%  [-14.9, -20.4] | 0.24 | -6.7%  [-3.9, -9.5] | 0.49 | -6.7%  [-3.9, -9.5] | 0.48 |
|  | Obese  (n=255) | -22.3%  [-18.0, -26.6] | 0.07 | -22.2%  [-18.0, -26.5] | 0.06 | -16.3%  [-12.1, -20.5] | 0.12 | -16.2%  [-12.1, -20.4] | 0.11 | -7.2%  [-2.9, -11.5] | 0.77 | -7.2%  [-2.8, -11.5] | 0.76 |

**Supplementary table 3: Percentage change in uterine artery pulsatility index over the course of gestation by fetal sex and maternal BMI category, expressed as percentage drop of Doppler PI between scanning timepoints.** CI; Confidence Interval. ^a^Model adjusted for gestational age at all scanning timepoints ^b^Model adjusted for gestational age at all scanning timepoints, maternal BMI, systolic blood pressure at 12 weeks gestation, marital status, maternal age, maternal ethnicity, maternal smoking status and deprivation index. ^c^p-value relative to mean uterine artery pulsatility index drop in normal weight women at same scanning timepoint.
