# Supplementary material for: Independent influences of maternal obesity and fetal sex on maternal cardiovascular adaptation to pregnancy: a prospective cohort study
Source: Int J Obes (Lond). 2020 Jun 15;44(11):2246–55. doi: 10.1038/s41366-020-0627-2 (PMC7577853; doi:10.1038/s41366-020-0627-2)
Supplement: Supplementary file 4 — Supplementary table 4 [file 41366_2020_627_MOESM4_ESM.docx]

|  | Drop between 20- and 36-week scan | | | | Drop between 20- and 28-week scan | | | | Drop between 28- and 36-week scan | | | |
| --- | --- | --- | --- | --- | --- | --- | --- | --- | --- | --- | --- | --- |
|  | Model 1^a^ | | Model 2^b^ | | Model 1^a^ | | Model 2^b^ | | Model 1^a^ | | Model 2^b^ | |
|  | Percentage decrease  [95 % CI] | p value^c^ | Percentage decrease  [95 % CI] | p value^c^ | Percentage decrease  [95 % CI] | p value^c^ | Percentage decrease  [95 % CI] | p value^c^ | Percentage decrease  [95 % CI] | p value^c^ | Percentage decrease  [95 % CI] | p value^c^ |
| Normal weight  (n=2164) | -31.2%  [-30.3, -32.1] | ref | -31.2%  [-30.3, -32.1] | ref | -16.8%  [-15.9, -17.7] | ref | -16.8%  [-15.9, -17.7] | ref | -17.3%  [-16.4, -18.2] | ref | -17.3%  [-16.4, -18.2] | ref |
| Overweight  (n=1059) | -32.2%  [-30.9 -33.6] | 0.06 | -32.2%  [-30.9 -33.6] | 0.06 | -16.4%  [-15.1, -17.7] | 0.54 | -16.4%  [-15.1, -17.7] | 0.54 | -18.9%  [-17.6, -20.2] | 0.01 | -18.9%  [-17.6, -20.2] | 0.01 |
| Obese  (n=519) | -31.9%  [-30.0, -33.8] | 0.32 | -31.9%  [-30.0, -33.8] | 0.32 | -15.7%  [-13.8, -17.6] | 0.22 | -15.7%  [-13.8, -17.6] | 0.22 | -19.2%  [-17.3, -21.2] | 0.03 | -19.2%  [-17.3, -21.2] | 0.03 |

**Supplementary table 4:** **Percentage change in umbilical artery pulsatility index over the course of gestation by maternal BMI category, expressed as percentage drop of Doppler PI between scanning timepoints.** CI; Confidence Interval. ^a^Model adjusted for gestational age at all scanning timepoints ^b^Model adjusted for gestational age at all scanning timepoints, maternal BMI, systolic blood pressure at 12 weeks gestation, marital status, maternal age, maternal ethnicity, maternal smoking status and deprivation index. ^c^p-value relative to mean umbilical artery pulsatility index drop in normal weight women at same scanning timepoint.
