# Supplementary material for: Independent influences of maternal obesity and fetal sex on maternal cardiovascular adaptation to pregnancy: a prospective cohort study
Source: Int J Obes (Lond). 2020 Jun 15;44(11):2246–55. doi: 10.1038/s41366-020-0627-2 (PMC7577853; doi:10.1038/s41366-020-0627-2)
Supplement: Supplementary file 5 — Supplementary table 5 [file 41366_2020_627_MOESM5_ESM.docx]

|  |  | Drop between 20- and 36-week scan | | | | Drop between 20- and 28-week scan | | | | Drop between 28- and 36-week scan | | | |
| --- | --- | --- | --- | --- | --- | --- | --- | --- | --- | --- | --- | --- | --- |
|  |  | Model 1^a^ | | Model 2^b^ | | Model 1^a^ | | Model 2^b^ | | Model 1^a^ | | Model 2^b^ | |
|  |  | Percentage decrease  [95 % CI] | p value^c^ | Percentage decrease  [95 % CI] | p value^c^ | Percentage decrease  [95 % CI] | p value^c^ | Percentage decrease  [95 % CI] | p value^c^ | Percentage decrease  [95 % CI] | p value^c^ | Percentage decrease  [95 % CI] | p value^c^ |
| Normal weight | Male fetus  (n=1090) | -30.5%  [-29.2, -31.7] | ref | -30.5%  [-29.2, -31.7] | ref | -16.5%  [-15.3, -17.8] | ref | -16.5%  [-15.3, -17.8] | ref | -16.7%  [-15.5, -18.0] | ref | -16.7%  [-15.5, -18.0] | ref |
|  | Female fetus  (n=1074) | -32.0%  [-30.7, -33.2] | 0.02 | -32.0%  [-30.7, -33.2] | 0.02 | -17.1%  [-15.9, -18.4] | 0.40 | -17.1%  [-15.9, -18.4] | 0.40 | -17.9%  [-16.6, -19.2] | 0.13 | -17.9%  [-16.6, -19.2] | 0.13 |
| Overweight | Male fetus  (n=531) | -31.0%  [-29.1, -32.8] | ref | -31.0%  [-29.1, -32.9] | ref | -15.9%  [-14.1, -17.7] | ref | -15.9%  [-14.1, -17.7] | ref | -17.9%  [-16.0, -19.8] | ref | -17.9%  [-16.0, -19.8] | ref |
|  | Female fetus  (n=528) | -33.5%  [-31.6, -35.3] | 0.006 | -33.5%  [-31.6, -35.3] | 0.006 | -16.9%  [-15.1, -18.7] | 0.36 | -16.9%  [-15.1, -18.7] | 0.36 | -19.9%  [-18.1, -21.8] | 0.06 | -19.9%  [-18.1, -21.8] | 0.06 |
| Obese | Male fetus  (n=264) | -31.3%  [-28.5, -34.0] | ref | -31.3%  [-28.5, -34.0] | ref | -16.6%  [-13.8, -19.3] | ref | -16.6%  [-13.8, -19.3] | ref | -17.6%  [-14.8, -20.4] | ref | -17.6%  [-14.8, -20.4] | ref |
|  | Female fetus  (n=255) | -32.7%  [-29.8, -35.5] | 0.31 | -32.6%  [-29.8, -35.5] | 0.32 | -14.9%  [-12.1, -17.7] | 0.30 | -14.9%  [-12.1, -17.7] | 0.30 | -20.9%  [-18.0, -23.8] | 0.04 | -20.9%  [-18.0, -23.7] | 0.04 |

**Supplementary table 5: Percentage change in umbilical artery pulsatility index over the course of gestation by maternal BMI category and fetal sex, expressed as percentage drop of Doppler PI between scanning timepoints.** CI; Confidence Interval. ^a^Model adjusted for gestational age at all scanning timepoints ^b^Model adjusted for gestational age at all scanning timepoints, maternal BMI, systolic blood pressure at 12 weeks gestation, marital status, maternal age, maternal ethnicity, maternal smoking status and deprivation index. ^c^p-value relative to mean umbilical artery pulsatility index drop in normal weight women at same scanning timepoint.
