# Supplementary material for: Independent influences of maternal obesity and fetal sex on maternal cardiovascular adaptation to pregnancy: a prospective cohort study
Source: Int J Obes (Lond). 2020 Jun 15;44(11):2246–55. doi: 10.1038/s41366-020-0627-2 (PMC7577853; doi:10.1038/s41366-020-0627-2)
Supplement: Supplementary file 8 — Supplementary table 8 [file 41366_2020_627_MOESM8_ESM.docx]

|  | Drop between 20- and 36-week scan | | | | Drop between 20- and 28-week scan | | | | Drop between 28- and 36-week scan | | | |
| --- | --- | --- | --- | --- | --- | --- | --- | --- | --- | --- | --- | --- |
|  | Model 1^a^ | | Model 2^b^ | | Model 1^a^ | | Model 2^b^ | | Model 1^a^ | | Model 2^b^ | |
|  | Percentage decrease  [95 % CI] | p valuec^c^ | Percentage decrease  [95 % CI] | p valuec^c^ | Percentage decrease  [95 % CI] | p valuec^c^ | Percentage decrease  [95 % CI] | p valuec^c^ | Percentage decrease  [95 % CI] | p valuec^c^ | Percentage decrease  [95 % CI] | p valuec^c^ |
| Normal weight  (n=1906) | -25.5%  [-24.1, -27.0] | ref | -25.5%  [-24.1, -27.0] | ref | -19.5%  [-18.1, -20.9] | ref | -19.5%  [-18.1, -20.9] | ref | -7.5%  [-6.1, -8.9] | ref | -7.5%  [-6.1, -8.9] | ref |
| Overweight  (n=856) | -24.2%  [-22.1, -26.4] | 0.19 | -24.2%  [-22.1, -26.4] | 0.19 | -17.5%  [-15.4, -19.6] | 0.06 | -17.5%  [-15.4, -19.6] | 0.06 | -8.2%  [-6.0, -10.3] | 0.58 | -8.2%  [-6.0, -10.3] | 0.58 |
| Obese  (n=361) | -21.2%  [-17.8, -24.7] | 0.003 | -21.2%  [-17.7, -24.7] | 0.003 | -16.2%  [-12.8, -19.7] | 0.03 | -16.2%  [-12.8, -19.7] | 0.03 | -5.9%  [-2.5, -9.4] | 0.37 | -5.9%  [-2.4, -9.4] | 0.37 |

**Supplementary table 8: Sensitivity analysis of the effect of excluding women who developed gestational diabetes, preeclampsia, gestational hypertension or experienced preterm birth on the percentage change in uterine artery pulsatility index over the course of gestation by maternal BMI category, expressed as percentage drop of Doppler PI between scanning timepoints.** CI; Confidence Interval. ^a^Model adjusted for gestational age at all scanning timepoints ^b^Model adjusted for gestational age at all scanning timepoints, maternal BMI, systolic blood pressure at 12 weeks gestation, marital status, maternal age, maternal ethnicity, maternal smoking status and deprivation index. ^c^p-value relative to mean uterine artery pulsatility index drop in normal weight women at same scanning timepoint.
